# *Solidago canadensis* as a bioaccumulator and phytoremediator of Pb and Zn

**DOI:** 10.1007/s11356-019-06690-x

**Published:** 2019-11-19

**Authors:** Aleksandra Bielecka, Elżbieta Królak

**Affiliations:** Faculty of Natural Sciences, Siedlce University of Natural Sciences and Humanities, Prusa 14, 08-110 Siedlce, Poland

**Keywords:** Canadian goldenrod, Aboveground parts, Belowground parts, Soil, Heavy metals, Bioconcentration factor (BCF), Translocation factor (TF)

## Abstract

Canadian goldenrod (*Solidago canadensis* L.) is a plant that grows in a variety of environmental conditions. It shows high capability to spread in various habitats, including fallow lands and brownfield land. The research aimed at analyzing the content of Pb and Zn in the underground (roots, rhizomes) and aboveground parts (stems, leaves, inflorences) of *Solidago canadensis* (SC) originating from two locations that are clearly different in terms of metal content in soil. Statistically significant differences were determined in the content of Pb and Zn in soil and particular morphological parts of the plant, depending on the sampling location. It has been shown that in the conditions of increased (compared with natural) Pb and Zn content in the soil, SC may serve as a bioaccumulator of these metals. It was determined that SC can be used as a phytostabilizer of Pb and Zn in soils heavily contaminated with these elements. The content of Zn in the aboveground parts of SC indicates that this plant can also be used for phytoextraction of soils contaminated with this metal.

## Introduction

In recent years, plants of the genus *Solidago* (Asteraceae) have been given a relatively high level of attention in environmental studies. Goldenrods (*Solidago* spp.) are classified as invasive plants (Weber and Schmid 1998; Tokarska-Guzik et al. 2012). The native range of *Solidago* sp. is North America (Weber and Jacobs 2005). In the last century, *Solidago* species have spread throughout almost all of Europe (Guzikowa and Maycock 1986; Weber 1997, 2000) and Asia (Lu et al. 2007; Semple and Rao 2017). They also occur in Africa (Cheek and Semple 2016) and Australia (Semple and Uesugi 2017). The ability of *Solidago canadensis* L. (SC) to quickly colonize new areas results from the strong reproductive capacity of its underground parts, fast spreading and germination of seeds (Huang and Guo 2005). The species tolerates a wide range of environmental conditions (e.g., Jin et al. 2004; Huang et al. 2007). The species forms extensive and compact patches, especially in anthropogenic sites. It occurs in abandoned cultivated fields (Szymura and Wolski 2006) as well as on industrial wastelands (Vega et al. 2004; Antonijevic et al. 2012; Patrzałek et al. 2012). Individual clones form dense clusters, composed of many ramets, depending on the age of a given clone. The roots branch out from the base of ramets and reach the minimum depth of 20 cm (Weber 2000). The goldenrod also produces large amounts of aboveground biomass, estimated at about 20 Mg of fresh weight/ha in the case of 70% ground cover (Patrzałek et al. 2012). The ratio of the under- to aboveground biomass of SC is often between 0.25 and 0.82 (Wang et al. 2017).

Many scientific papers relate to the impact of SC on biodiversity decline (e.g., Huang and Guo 2005; Gioria and Osborne 2014; Fenesi et al. 2015; Holeksa et al. 2015). Literature, however, lacks comprehensive information on the content of metals, including Pb and Zn, in particular morphological parts of the plant. Studies aimed at assessing the accumulation of heavy metals in the tissues of the plant (e.g., Vega et al. 2004; Huang et al. 2007; Yang et al. 2007a), but there is no data on the possibility of its use for phytoremediation. Due to the extensive underground system and its high biomass (Wang et al. 2017) and the role of rhizome as a storage organ in the plant, we have undertaken this study to assess the level of Pb and Zn accumulation in individual parts of SC occurring in two areas: agricultural and industrial.

The ability of plants to accumulate heavy metals is also used in biomonitoring of the environment (Markert et al. 2003). Biomonitoring studies including the analysis of bioaccumulation of metals in plants are also undertaken to identify species that can be used in phytoremediation (Massa et al. 2010). Plants used in phytoremediation should be capable to colonize contaminated areas and should be adaptive to edaphic conditions. They should characterize with the ease of taking heavy metals from the environment. Phytostabilization processes are connected with the reduction of bioavailability and mobility of metals. Plants used in phytostabilization, in addition to their ability to retain metals in the roots, should have an extensive root systems. Phytoextration is connected with the uptake of a contaminant by plant roots from the environment and its translocation into harvestable plant biomass. Plant features that predispose them to phytoextraction include: high metal accumulation in aboveground parts, high biomass of aboveground parts, ease of harvesting, good adaptation to environmental and climatic conditions (Laghlimi et al. 2015).

Some traits of SC, such as wide range of tolerance to physicochemical conditions, ability to colonize contaminated soils, high biomass of aboveground parts, extensive underground system, the possibility of accumulation of heavy metals, and easy collection from the environment indicate the usefulness of the plant in both biomonitoring and phytoremediation.

Biomonitoring studies using SC as a heavy metal accumulator are conducted to a limited extent. Few reports refer to Pb content in plant roots (Xiang et al. 2010; Yang et al. 2008), Pb and Zn content in stems, leaves and roots (Antonijevic et al. 2012). There is no complex information in the literature on the content of metals, including Pb and Zn, in all individual morphological parts of the plant, including inflorescences and rhizomes.

The objective of this study was to assess the content of Pb and Zn in different fully developed morphological parts (roots, rhizomes, stems, leaves, inflorescences) of SC originating from agricultural and industrial areas, different in terms of Pb and Zn content in the soil.

The following hypotheses were put forward in the paper:

- SC accumulates Pb and Zn in concentrations proportional to the content of metals in soil,
- Morphological parts of SC originating from agricultural and industrial areas differ in the content of Pb and Zn.

The possibility of using SC as a bioaccumulator and phytoremediator of both heavy metals was presented.

## Material and methods

### Study area

**Fig. 1:**
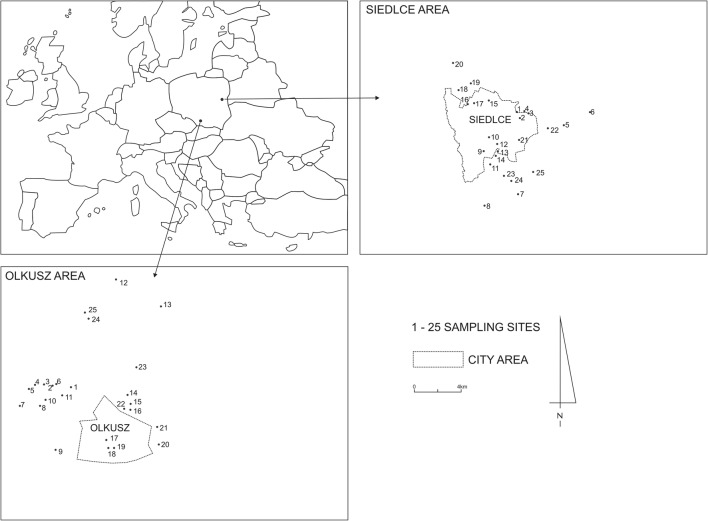
Location of the study area and sampling sites (Siedlce–agricultural area AA, Olkusz–industrial area IA)

The research was conducted in Poland in two locations: agricultural (AA–the region of Siedlce–central-eastern Poland: 52° 13′ 72″ N–52° 74′ 25″ N and 22° 13′ 29″ E–22° 25′ 24″ E) and industrial (IA–Olkusz–southern Poland: 50° 15′ 83″ N–50° 23′ 39″ N and 19° 26′ 50″ E–19° 36′ 46″ E) (Fig. 1). Podzolic and pseudopodzolic soils, developed on sand and sandy loam, dominate in the agricultural region. They are characterized by acid reaction, low humus content (Hoser and Pirowski 2014), and natural Pb and Zn content (Siebielec 2017).

The soil in the industrial region is characterized by high content of heavy metals. In the area, there are sites with extreme contents of Pb and Zn, which results from centuries-long industrial activity involving the extraction and processing of zinc and lead ores (Gruszecka and Wdowin 2013; Kapusta et al. 2015). In the region, loamy sand rich in calcium prevails (Wach et al. 2014).

### Plant material

Samples were collected from both locations in the last decade of August 2017 during intensive flowering of the Canadian goldenrod. The sites were selected at random; their choice was based on the presence of the goldenrod at an area of at least 500 m^2^. Twenty five sites were selected at each location. At each site, three plots with an area of 9 m^2^ (3 × 3 m) were selected, located at least 2 m from the edge of a given site and diagonally across the examined surface. The number of ramets were counted at each plot to determine the density of the goldenrod. Next, the goldenrod samples were collected from each plot, using a 40 × 40 cm frame (0.16 m^2^). The aboveground (stems, leaves, inflorescences) and underground (roots and rhizomes) parts of plants were cut—the latter from a depth of up to 20 cm. Ramets cut from the surface of 0.16 m^2^ were counted and used to determine the biomass of the aboveground parts of the plant per 1 ramet. The collected plants were healthy, without any sign of morphological damage. Twenty ramets were sampled for analysis from each site. Leaves, stems and inflorescences, roots, and rhizomes were isolated from each plant in the laboratory. The underground parts were carefully washed under running water and then rinsed in distilled water. The plant material was dried at a temperature of 60 °C to a constant weight. The mass of each morphological part per ramet was calculated following the determination of dry weight. The samples were homogenized. 1 g subsamples were pre-mineralized in a muffle furnace at a temperature of 420 °C, which was followed by microwave mineralization in 68% nitric acid (ultra-pure) and 30% hydrogen peroxide (ultra-pure) (3:2, v/v) (Ostrowska et al. 1991). Three independent subsamples were prepared. Mean values of the results were used in calculations prepared for each site.

### Soil samples

Four soil subsamples were collected in the field from each plot using Egner’s sampling stick. A total of 12 soil subsamples were collected from each site and pooled. They were representative samples for each site. Soil samples were air-dried in the laboratory. The soil was then sieved through a sieve with a mesh diameter of 2 mm. The representative soil subsamples were homogenized in an agate mortar, initially mineralized in a muffle furnace at a temperature of 420 °C and, as in the case of plants, mineralized in a mixture of HNO_3_ (68%) and H_2_O_2_ (30%) in a microwave mineralizer. Additionally, to determine the content of N, the soil samples (1 g) were mineralized in Kjeldahl flasks in 95% H_2_SO_4_ and 30% H_2_O_2_ (3:1, v/v) (Ostrowska et al. 1991).

### Analysis of heavy metals content in the plant material and soil samples

The content of lead and zinc in solutions obtained after mineralization was determined using the atomic absorption spectrometric (AAS) technique (an apparatus manufactured by Carl Zeiss Jena) and an acetylene-air flame. Standard solutions within the following ranges of concentration (μg ml^−1^): Pb 0.2–3.0 and Zn 0.3–3.6 were used for the determination of the metals. In the case of high content of metals in the samples, exceeding the concentration range of standard solutions, the examined samples were diluted. The reference material (INCP–PVTL–6) was used in the study, which was prepared and certified at the Institute of Nuclear Chemistry and Technology (Warsaw, Poland). Recoveries of lead and zinc were 104.5% and 96.7%, respectively, and the accuracy of measurements expressed by the coefficient of variation was 6.7% for Pb and 4.9% for Zn.

Results of the analysis of selected soil chemical parameters and the content of metals in individual morphological parts of the plant were calculated per 1 g DW of a given sample. Bioconcentration factors (BCF) were calculated based on the ratio of the concentration of the metal in the root and the concentration of that metal in the soil, while translocation factors (TF) were calculated as the ratio of metal concentration in rhizomes, stems, leaves, and flowers to the metal concentration in the root (Malik et al. 2010).

### Analysis of soil reaction, the contents of organic carbon, and nitrogen

The soil reaction was measured in 1 M KCl (1:2.5), the content of organic carbon was determined with the use of Tiurin’s method (Ostrowska et al. 1991) and nitrogen—with the use of indophenol method (Marczenko 1979).

#### Statistical analysis

The normality of data was tested using the Shapiro-Wilk test and homoscedasticity with the Brown-Forsythe test. Non-parametric tests were used because of non-normal distributions of the variables and heteroscedasticity. The Mann-Whitney test was applied to compare soil acidity, the content of organic carbon, the content of nitrogen, and the content of Pb and Zn in the soil and in parts of the plant in both locations, while the significance of differences in the content of metals in different morphological parts of the plant and in soil samples in the same location was calculated using Kruskal-Wallis test and Dunn’s post-hoc test. Spearman’s correlation rank coefficients were used as a measure of the strength of the relationships between the content of Pb and Zn in the soil and in the plant parts. The similarity (based on Euclidean distance) between the study sites in terms of Pb and Zn content in the soil and SC tissues was analyzed using the clustering with Ward’s linkeage method. To compare the values of the number of ramets per 1 m^2^ and the biomass of particular morphological parts, *t* test was used. Statistical analyses were performed using STATISTICA 12 software.

## Results

Soil samples collected in the agricultural area are characterized with lower pH, organic carbon, and nitrogen contents compared with the samples collected in the industrial area (Table 1).

**Table 1:** Soil reaction, organic carbon and nitrogen content in soil samples collected in agricultural (AA) and industrial (IA) areas (*N* = 50)

Parameter	Unit	AA				IA				M-W test value
		Mean	Median	SD	Range	Mean	Median	SD	Range	
Acidity	pH	5.03	4.84	0.99	3.88–7.51	6.51	6.53	0.77	5.00–7.75	*Z* = 4.49, *p* < 0.001
Corg	%	1.66	1.54	0.47	1.03–2.64	2.69	2.19	1.64	1.28–9.70	*Z* = 3.90, *p* < 0.001
N	mg/kg	1.01	0.91	0.28	0.63–1.79	1.36	1.16	0.79	0.52–4.62	*Z* = 2.41, *p* = 0.016

Soil samples in the selected locations were significantly different in terms of Pb and Zn content. The content of Pb and Zn in the soil samples from the industrial area described by the median value was about 10 times higher compared with the soil samples from the agricultural area (Table 2).

**Table 2:** The content of Pb and Zn (mg kg^−1^) in soil and morphological parts of *Solidago canadensis* at the sites in agricultural (AA) and industrial areas (IA). Significant differences in the same types of samples from both locations were checked with the Mann-Whitney (M-W) test (*N* = 50). Indices a, b, c, d, e, and f show significant differences among different types of samples from each location checked with the Kruskal-Wallis test (*N* = 25, *p* < 0.001) followed by Dunn’s test. The value of the Kruskal-Wallis test (*p* < 0.0001): Pb, AA–H_5,150_ = 131.63, IA–H_5,150_ = 130.88; Zn: AA–H_5,150_ = 67.00, IA–H_5,150_ = 81.60

Sample	Pb					Zn				
	AA		IA		M-W test (*Z*; *p*)	AA		IA		M-W test (*Z*; *p*)
	Median	Range	Median	Range		Median	Range	Median	Range	
Soil	22.8^a,c,e^	17.4–40.2	201.0^a,b^	69.5–1626.5	6.05; *p* = 0.000	42.0^a,b,c,d^	11.2–125.9	349.7^a,b,e^	85.4–11626.1	5.98; *p* = 0.000
Roots	23.3^b,e^	19.2–27.8	83.4^a,b^	38.5–789.5	6.02; *p* = 0.000	28.9^a,b,c^	2.9–343.5	441.8^a,b^	221.1–2227.0	5.87; *p* = 0.000
Rhizomes	11.0^a,c,f^	5.5–16.5	27.0^c,e^	15.4–346.6	5.39; *p* = 0.000	38.9^a,b,c,d,e^	16.4–155.0	140.3^c,d,e^	39.5–2163.8	4.81; *p* = 0.000
Stems	5.75^d,f^	4.4–7.7	9.2^d,f^	7.7–23.9	3.26; *p* = 0.001	85.8^a,c,d,e^	19.5–489.2	106.4^c,d,e^	36.0–431.2	ns
Leaves	18.9 ^a,b,e^	12.4–21.1	32.5^c,e^	25.9–55.8	5.18; *p* = 0.000	66.9^c,d,e^	26.5–492.0	164.4^a,c,d,e^	48.2–969.8	2.73; *p* = 0.006
Inflorescences	7.2^c,d,f^	5.3–8.9	13.0^d,f^	10.1–22.1	5.66; *p* = 0.000	19.0^f^	12.4–35.3	45.2^f^	29.2–332.0	5.98; *p* = 0.000

*ns*, not significant

The accumulation of Pb per 1 kg DW in particular parts of the plant decreased in the following order: IA–root > rhizome > leaves > inflorescence > stem and AA: root > leaves > rhizome > inflorescence > stem. The accumulation of Zn in specific morphological parts of the plant calculated per 1 kg DW varied as follows: IA: roots > rhizomes > leaves > stems > inflorescences, AA: stems > leaves > rhizomes > roots > inflorescences. The content of both metals in different morphological parts of SC collected in IA was significantly higher than in AA (Table 2).

The co-occurrence of Zn and Pb in the soil was determined in both locations. Statistically significant correlations between Zn content in the soil and Zn content in the roots, stems, leaves, and inflorescences of SC, as well as between Pb content in the soil and Pb content in the roots, rhizomes, stems, and leaves of SC were found in the goldenrod samples from the industrial area. No similar correlations were between the content of the metals in the soil and tissues of SC harvested at the agricultural sites (Table 3).

**Table 3:** Significant Spearman’s rank correlation coefficients between Pb and Zn in the soil samples and morphological parts of *Solidago canadensis* in the agricultural and industrial areas; abbreviations in table: S, soil; R, roots; Rh, rhizomes; St, stems; L, leaves; I, inflorescences. Significant results are in bold (*p < 0.05, **p < 0.01, ***p < 0.001)

Metal	Sample	Pb						Zn					
		S	R	Rh	St	L	I	S	R	Rh	St	L	I
Agricultural area													
Pb	S	-											
	R	− 0.05	-										
	Rh	− 0.05	− 0.26	-									
	St	− 0.04	− 0.84***	0.28	-								
	L	− 0.20	0.06	0.11	0.04	-							
	I	0.42*	− 0.17	0.30	− 0.02	− 0.32	-						
Zn	S	0.92***	**-**					-					
	R	− 0.07	0.18	-				0.14	-				
	Rh	− 0.02	− 0.16	0.01	-			− 0.03	0.55**	-			
	St	0.03	− 0.22	0.11	− 0.36	-		− 0.01	0.45*	0.70***	-		
	L	− 0.14	− 0.42*	0.15	− 0.14	−0.24	-	− 001	0.34	0.62***	0.67***	-	
	I	0.34	− 0.12	− 0.05	− 0.13	0.14	− 0.04	**−** 0.06	0.32	0.42*	0.42*	0.58**	-
Industrial area													
Pb	S	-											
	R	0.60**	-										
	Rh	0.43*	0.65***	-									
	St	0.58**	0.27	0.32	-								
	L	0.51**	0.11	0.19	0.33	-							
	I	0.39	0.11	0.34	0.50*	0.38	-						
Zn	S	0.86***	**-**					-					
	R	0.60**	0.57**	**-**				0.58**	-				
	Rh	**0.34**	0.40*	0.70***	**-**			0.38	0.66***	-			
	St	0.52**	0.25	0.39	0.56**	**-**		0.42*	0.73***	0.70***	-		
	L	0.67***	0.28	0.31	0.51**	0.25	-	0.62***	0.64***	0.60**	0.80***	-	
	I	0.52**	0.28	0.36	0.62**	0.36	0.68***	0.52**	0.59**	0.50*	0.68***	0.73***	-

The correlation analysis showed no effect of selected soil parameters (pH, organic carbon, and nitrogen content) on the metal content in the examined parts of SC. The only significant correlation (*p* < 0.01) between soil pH on Pb and Zn content was noted in soil samples from the agricultural area (*R*_s_ = 0.59 and *R*_s_ = 0.72 respectively). A significant correlation (*p* < 0.05) was also noted between organic carbon content and Pb and Zn contents in samples taken in both locations (*R*_s_ values in the range 0.41–0.51).

Values of the Pb bioconcentration factor in the root/soil system as well as of the factor of Pb translocation from roots to other morphological parts of the plant were higher in the agricultural area than in the industrial area. Similarly, higher values of Zn translocation factor from roots to the rhizomes, stems, leaves, and flowers were determined in goldenrod harvested in the agricultural area (Table 4).

**Table 4:** Values of Pb and Zn bioconcentration (BCF) and translocation (TF) factors calculated for samples collected in agricultural (AA) and industrial (IA) areas. Significant differences in the same types of samples from both locations were checked with the Mann-Whitney (M-W) test (*N* = 50); abbreviations as in Table 3

	System	Pb		M-W test (*Z*; *p*)	Zn		M-W test (*Z*; *p*)
		AA	IA		AA	IA	
BCF	R/S	1.01 ± 0.21	0.56 ± 0.27	*Z* = 4.79; *p* < 0.001	1.24 ± 1.43	1.70 ± 1.27	*Z* = 2.13; *p* < 0.05
TF	Rh/R	0.48 ± 0.14	0.35 ± 0.15	*Z* = 2.05; *p* < 0.01	0.69 ± 0.80	0.31 ± 0.19	*Z* = 5.86; *p* < 0.001
	St/R	0.26 ± 0.06	0.11 ± 0.05	*Z* = 5.70; *p* < 0.001	4.14 ± 4.21	0.26 ± 0.14	*Z* = 5.59; *p* < 0.001
	L/R	0.81 ± 0.11	0.39 ± 0.22	*Z* = 5.30; *p* < 0.001	4.31 ± 4.47	0.37 ± 0.19	*Z* = 3.32; *p* < 0.001
	I/R	0.32 ± 0.04	0.16 ± 0.09	*Z* = 5.30; *p* < 0.001	0.81 ± 0.84	0.11 ± 0.05	*Z* = 5.61; *p* < 0.001

**Fig. 2:**
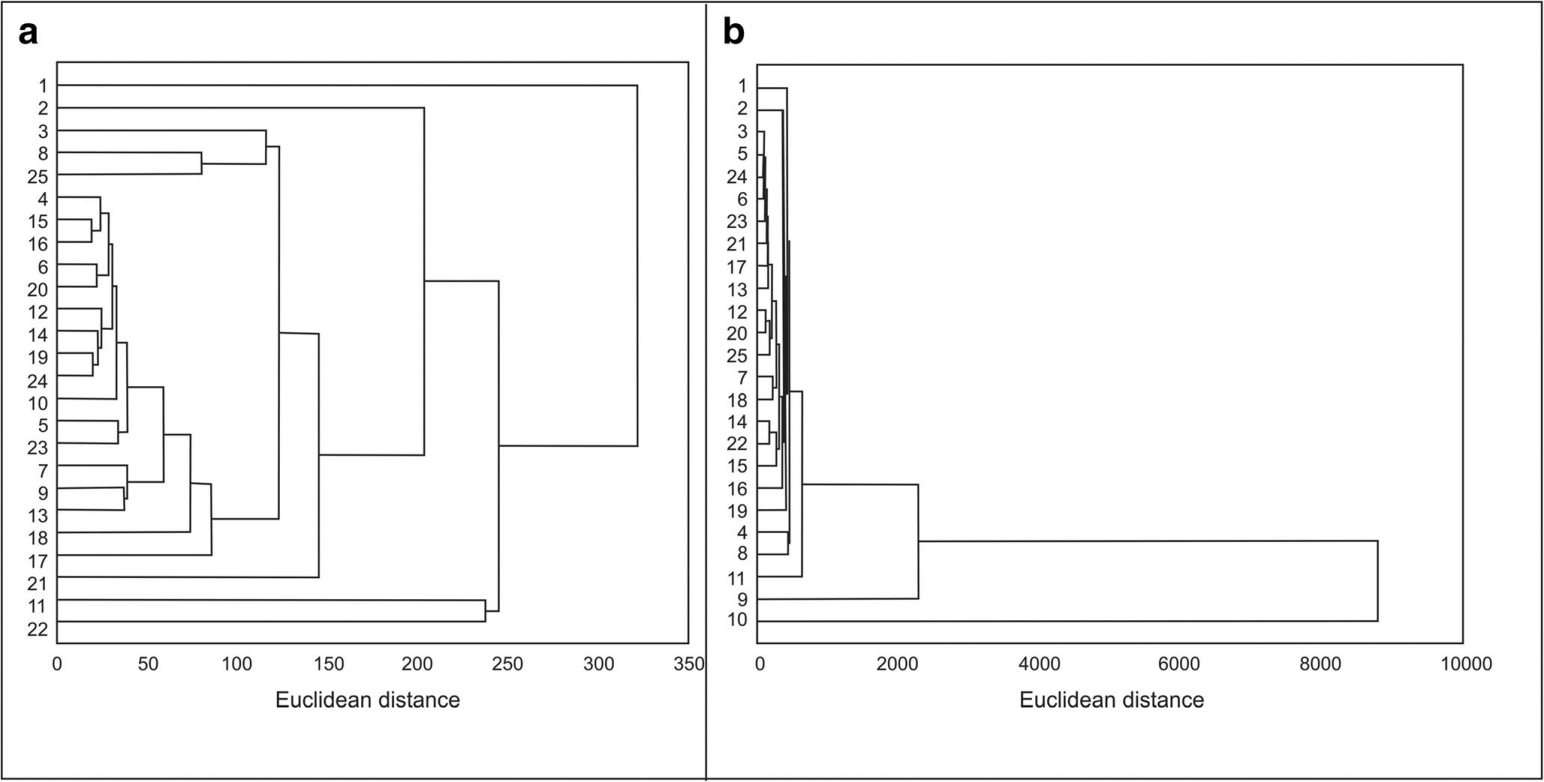
Results of dendrological analysis presenting the similarity in the Pb and Zn content in soil and morphological parts of *Solidago canadensis* at the sites located in agricultural and in industrial areas

Cluster analysis showed a greater similarity of samples in terms of Pb and Zn content in the agricultural (Euclidean distance below 350; Fig. 2A) than in the industrial area (Euclidean distance above 8000; Fig. 2B). In the dendrogram presenting Pb and Zn contents in samples from the agricultural area (Fig. 2A), the extreme positions are occupied by sites with higher zinc (sites 1, 2) and lead (sites 11, 12) content. In the industrial area (Fig. 2B), samples taken from the sites located close to Bolesław Mining and Metallurgical Plants (sites 9–11) were distinguished by the highest Pb and Zn contents.

**Fig. 3:**
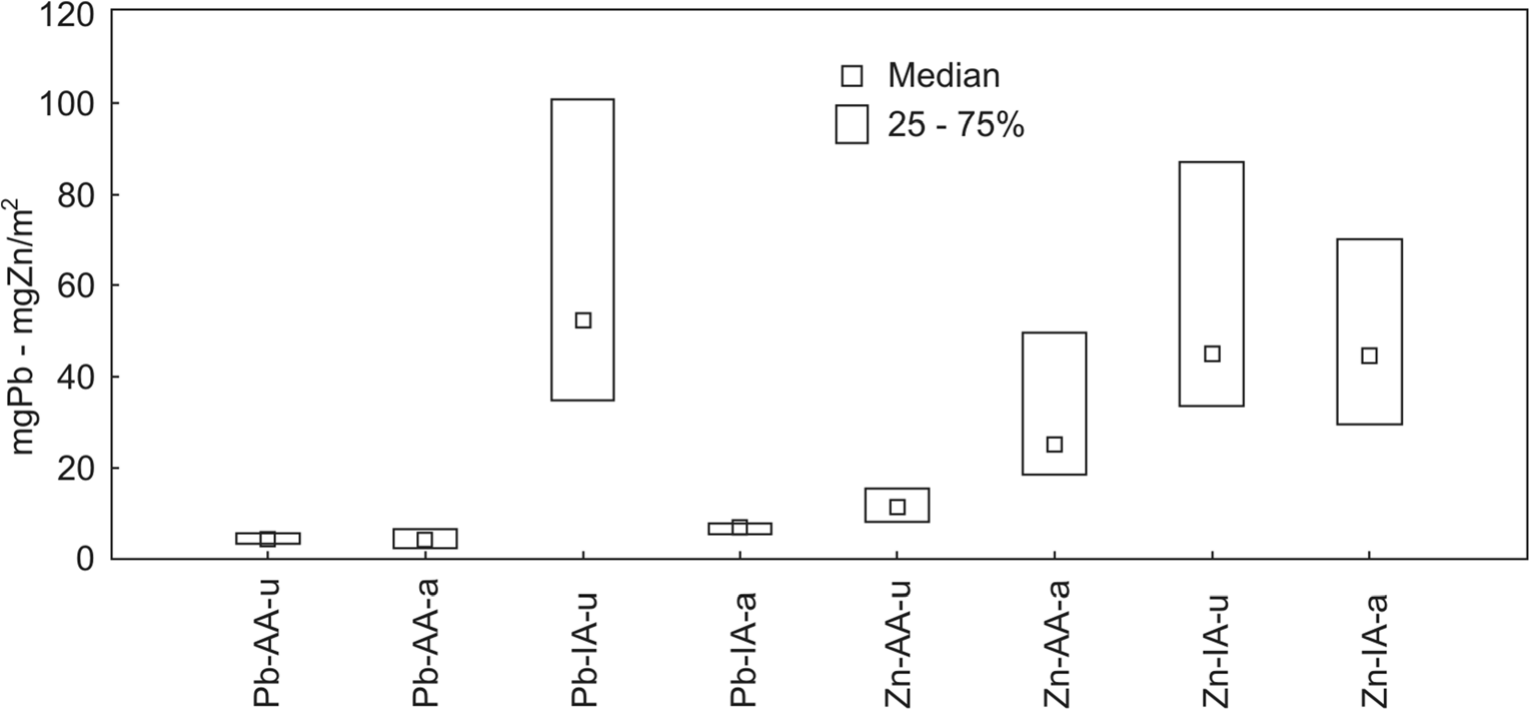
Accumulation of Pb and Zn in underground (u) and aboveground (a) parts of *Solidago canadensis* per m^2^ in the two locations: agricultural (AA) and industrial (IA) areas

The mean density of the goldenrod at the study sites in the agricultural area was c. 45 ± 18.3 ramets m^−2^, while in the industrial area – 36 ± 12.3 ramets m^−2^; these values were statistically significant (*t* = 2.03, *p* = 0.048, df = 48). The dry weight of aboveground and underground parts calculated per 1 ramet was given in Table 5. The average percentage contribution of individual morphological parts to the dry weight of SC harvested in agricultural and industrial areas was, respectively, as follows: roots, 12.7 and 8.7%; the rhizomes, 28.5 and 25.9%; the stems, 32.3 and 36.0%; leaves, 13.5 and 15.8%; flowers, 13.0 and 13.6%. The above results and the results concerning the content of Pb and Zn in different morphological parts of the plant were used to calculate the content of metals in the underground (u) and aboveground (a) parts of SC per m^2^ (Fig. 3).

**Table 5:** Dry matter (mean, median, SD) of specific morphological parts calculated per 1 ramet of *Solidago canadensis* in agricultural (AA) and industrial (IA) areas (*N* = 50, df = 48)

Part of the plant	AA				IA				*t* test value
	Mean	Median	SD	Range	Mean	Median	SD	Range	
Inflorescences	2.31	2.40	1.00	0.62–4.10	2.49	2.45	0.69	1.60–4.35	*t* = 0.72, *p* = 0.47
Leaves	2.38	2.23	0.90	0.76–4.30	2.90	2.80	0.91	1.15–4.85	*t* = 2.07, *p* = 0.04
Stems	5.70	5.25	2.57	1.36–11.5	6.58	6.24	1.90	4.01–10.7	*t* = 1.38, *p* = 0.17
Rhizomes	5.03	4.73	1.82	2.61–10.5	4.74	4.75	2.65	1.56–12.7	*t* = 0.44, *p* = 0.66
Roots	2.25	2.25	0.61	1.43–3.83	1.59	1.49	0.74	0.64–3.17	*t* = 3.43, *p* = 0.001

In the samples of goldenrod harvested in the industrial area, Pb accumulated in comparable amounts in the aboveground and underground parts of the plant, and in the industrial area the accumulation of Pb in the underground parts was more than seven times higher compared with the aboveground parts. On the other hand, the content of Zn in the aboveground parts of SC from the agricultural area was more than two times higher compared with the underground parts. In the industrial area, the zinc content in the above- and underground samples of goldenrod was similar (Fig. 3).

## Discussion

The presented results of the research indicate that the content of Pb and Zn in the soil and different morphological parts of *S. canadensis* clearly varies depending on the location of the study sites. The Pb and Zn content for most of the analyzed soil samples collected in the agricultural area (Siedlce region) are within the range of values considered to be a natural content of these metals in light soils of Poland (Siebielec 2017), i.e., 50 mg Pb kg^−1^ and 70 mg Zn kg^−1^ (Kabata-Pendias et al. 1995). The industrial area (Olkusz region) is characterized by a much higher content of Zn and Pb due to natural deposits of Zn and Pb ores and related long-term mining and processing industry. Kapusta et al. (2015) reported the following content in the Olkusz ore-bearing region: 93–33,178 mg Pb kg^−1^ and 132–72,089 mg Zn kg^−1^ of soil. Gruszecka and Wdowin (2013) reported the average content of Pb and Zn in soil near Olkusz as 922 mg Pb kg^−1^ and 694 mg Zn kg^−1^. In our research, the highest content of Pb and Zn in the Olkusz region was recorded in samples collected from the sites located near Zakłady Górniczo-Hutnicze ZGH Bolesław (Bolesław Mining and Metallurgical Plants). This area is characterized by the highest level of soil contamination with Pb and Zn in the entire region (Gruszecka and Wdowin 2013; Kapusta et al. 2015). The range of Pb and Zn content in soil determined in our research does not differ significantly from the values reported in the literature. In both locations, the content of both metals in soil were significantly correlated.

The presence of SC in both locations, including sites heavily contaminated with Pb and Zn, is consistent with literature data on the wide physiological tolerance of SC to environmental conditions (e.g., Jin et al. 2004; Huang et al. 2007) including the high content of heavy metals in soil (Huang et al. 2007; Yang et al. 2007b; Antonijevic et al. 2012; Xiang et al. 2010). Significant correlations between the concentration of contaminants in plant tissues and their concentration in the environment indicate the possible applicability of the plant as a biomonitor (Markert et al. 2003). In our study, significant correlations were determined between Pb and Zn content in different morphological parts of SC and the content of these metals in soil in the industrial area. However, no such relationships were found in the agricultural area. Based on the obtained data, it can be assumed that SC may serve as a bioaccumulator of Pb and Zn in a contaminated environment.

In both locations, roots of *S. canadensis* accumulate the largest amounts of Pb per unit mass. According to literature, in the environment with a high content of Pb in the soil, this element is accumulated mainly in the roots (Xiang et al. 2010), while Zn, as a more mobile element compared with Pb (Vaněk et al. 2005), is transported to the aboveground plant parts. In plants, Pb uptake causes toxic effects resulting in decrease of biomass production (Gupta et al. 2013). Roots have the ability to absorb significant amounts of Pb, while significantly reducing its translocation to the aboveground parts (Sharma and Dubey 2005). Lead which penetrates into the root symplast is detoxified in vacuoles, cell walls, and dictiosomal vesicles (Wierzbicka 1995). Limited translocation of Pb occurs from root to other parts due to the barrier function of the root endodermis (Sharma and Dubey 2005). Unlike lead, zinc plays a fundamental role in the functioning of plants, e.g., in maintenance of the integrity of cellular membranes, protein synthesis, and regulation of auxin. The metal plays a key role in both flower and normal fruit development (Hafeez et al. 2013).

The movement of metals from soil to plants is determined by specific soil properties, e.g., pH, the content of organic matter and the content of nutrients (e.g., Takáč et al. 2009; Bini et al. 2012). Our results did not show a significant relationship between selected physicochemical properties of soil and the accumulation of Pb and Zn in SC tissues. The lack of impact of selected soil indicators on the accumulation of metals in the underground and aboveground parts of the SC may be the result of the impact of *Solidago* itself on the soil. The presence of goldenrod not only changes the chemical parameters of soil (Lu et al. 2005; Vanderhoeven et al. 2006; Zhang et al. 2009) but it also causes a decrease in soil microbial activity (e.g., Zhang et al. 2009; Scharfy et al. 2010; Dong et al. 2015). A decrease in soil microbial activity also occurs in conditions of heavy soil contamination with heavy metals (Lee et al. 2002; Yang et al. 2007b). Under conditions of heavy soil contamination with heavy metals, metal accumulation in plant tissues decreases (Lee et al. 2002; Yang et al. 2007b). The results of our research indicate that the bioconcentration factor of Pb and translocation factor of Pb and Zn reach lower values in the environment with a higher content of metals in the soil. Similar relationships between the content of metals in soil contaminated with lead and zinc and their accumulation in plants were observed by Cook et al. (1994).

Based on the BCF and TF values, it is possible to assess the suitability of specific plant species for phytostabilization and phytoextraction (Ghosh and Singh 2005; Mleczek et al. 2017). The authors emphasize that plants with high BCF and low TF can be used for the phytostabilization of metals. In the case of Pb, the BCF values were higher compared with the TF values in both locations. In the case of Zn, the BCF values were higher compared with TF values in samples from the industrial area. It appears that SC can be used as a phytostabilizer of Pb and Zn in soils heavily contaminated with these metals.

In both locations, roots of *S. canadensis* accumulate the largest amounts of Pb, which is particularly visible in the plant harvested in the contaminated area. In the industrial region, SC accumulates on average about 70 g Pb ha^−1^ in the aboveground parts during the flowering season, and about 520 g Pb ha^−1^ in the underground parts. In the area with a natural content of Pb in the soil (agricultural area), the accumulation of Pb in the above- and underground parts is similar—about 40 g Pb ha^−1^ on average. Similar results concerning Pb accumulation in plants overgrowing old heaps associated with metallurgy industry received Stefanowicz et al. (2016). Plant species: *F. vesca*, *P. arenaria*, *P. lanceolata*, and *S. ochroleuca*, similarly as *S. canadensis*, accumulate the highest contents of Pb in the roots so they can be used in phytostabilization. Yang et al. (2008) point to the exceptionally high accumulation of Pb in the underground parts of *Solidago canadensis* compared with the aboveground parts. Also, Xiang et al. (2010) emphasize the role of SC roots as a Pb accumulator and pay attention to the relatively high (BCF >  1), compared with other metals, accumulation factor of Pb.

The results of the conducted research indicate that SC can be used as a phytoremediator of Zn in particular. In the industrial area, its accumulation in the aboveground and underground parts of plants is similar and on average amounts to approximately 450 g Zn ha^−1^. In the area with a natural Zn content in the soil, this metal is accumulated in larger amounts (approximately 250 g ha^−1^ on average) in the aboveground parts of SC compared with the underground parts (about 110 g ha^−1^ on average).

Our results regarding the accumulation of Pb and Zn in SC biomass obtained from 1 ha are comparable with the values measured for other plant species used in phytoremediation (*Sorghum biocolor*, *Helianthus annuus*, *Brassica juncea*, *Medicago sativa*, *Zea mays*) which fall within ranges: Pb, 16 g ha^−1^ (*H. annuus*)–380 g ha^−1^ (*S. biocolor*); Zn, 410 g ha^−1^ (*H. annuus*)–1410 g ha^−1^ (*S. biocolor*) (Zhuang et al. 2009 and other authors cited in their paper).

Apart from the high content of zinc in the aboveground parts, additional traits that predispose the plant for phytoremediation are the high biomass (Patrzałek et al. 2012) and the occurrence in industrial areas (Vega et al. 2004; Antonijevic et al. 2012; Patrzałek et al. 2012). The removal of the aboveground biomass during the flowering period of the plant and its use as an energy source can be an effective method in the process of ecological restoration of zinc-contaminated areas.

## Conclusions

The research on the accumulation of Pb and Zn in the above- and underground parts of SC occurring in the uncontaminated environment and in the region affected by the mining and processing of Pb and Zn ores, hence characterized by an extremely high content of these elements in the soil, showed that SC can be used as:

- a bioaccumulator of lead and zinc content in the environment with increased content of Pb and Zn in the soil,
- a phytostabilizer of Pb and Zn, mainly in contaminated soils. This is determined by the high accumulation of these metals in the underground parts of the plant,
- a phytoextractor of Zn-evidenced by a relatively high accumulation of this element in the aboveground parts of SC.
